# Prevention of Cyclophosphamide-Induced Immunosuppression in Mice With Traditional Chinese Medicine Xuanfei Baidu Decoction

**DOI:** 10.3389/fphar.2021.730567

**Published:** 2021-10-19

**Authors:** Huimin Yan, Jia Lu, Jiabao Wang, Lu Chen, Yu Wang, Lin Li, Lin Miao, Han Zhang

**Affiliations:** ^1^ State Key Laboratory of Component-based Chinese Medicine, Tianjin University of Traditional Chinese Medicine, Tianjin, China; ^2^ Institute of Traditional Chinese Medicine, Tianjin University of Traditional Chinese Medicine, Tianjin, China; ^3^ Key Laboratory of Pharmacology of Traditional Chinese Medical Formulae, Ministry of Education, Tianjin University of Traditional Chinese Medicine, Tianjin, China

**Keywords:** Xuanfei Baidu decoction, immunosuppression, immune modification, cyclophosphamide, traditional Chinese medicine

## Abstract

**Background and aims:** Xuanfei Baidu decoction (XFBD), a traditional Chinese medicine formulation, was designed and successfully applied for COVID-19 disease treatment in China, while the mechanism is still not clear.

**Methods:** To evaluate the protective effect of XFBD on immunosuppression in cyclophosphamide (CY)-treated mice, XFBD was orally administrated, the body weight was measured, and the immune organ index was calculated. HE staining was performed to analyze the pathological structures of the liver, spleen, and thymus. The levels of cytokines and immunoglobulin in the serum and spleen were evaluated by ELISA and RT-PCR. Splenic lymphocytes were isolated, and LPS-stimulated cell proliferation and the number of CD4^+^ and CD8^+^ T lymphocytes were evaluated.

**Results:** XFBD significantly suppressed body weight loss and increased the indices of spleen and thymus. The pathological alteration was much improved after XFBD administration. The reductions of TNF-α, IFN-γ, IgG, and IgM levels in serum and IL-2, IL-4, and IL-6 expressions in the spleen were all significantly alleviated by XFBD. Splenic lymphocyte proliferation in response to LPS was further enhanced after treatment with XFBD. The reduction of CD4^+^ and CD8^+^ T lymphocytes in CY-treated mice was also highly increased in XFBD groups.

**Conclusion:** Our findings suggested that XFBD played a crucial role in protection against immunosuppression in CY-treated mice and could be a potential candidate for immune modification and therapy.

**GRAPHICAL ABSTRACT:**
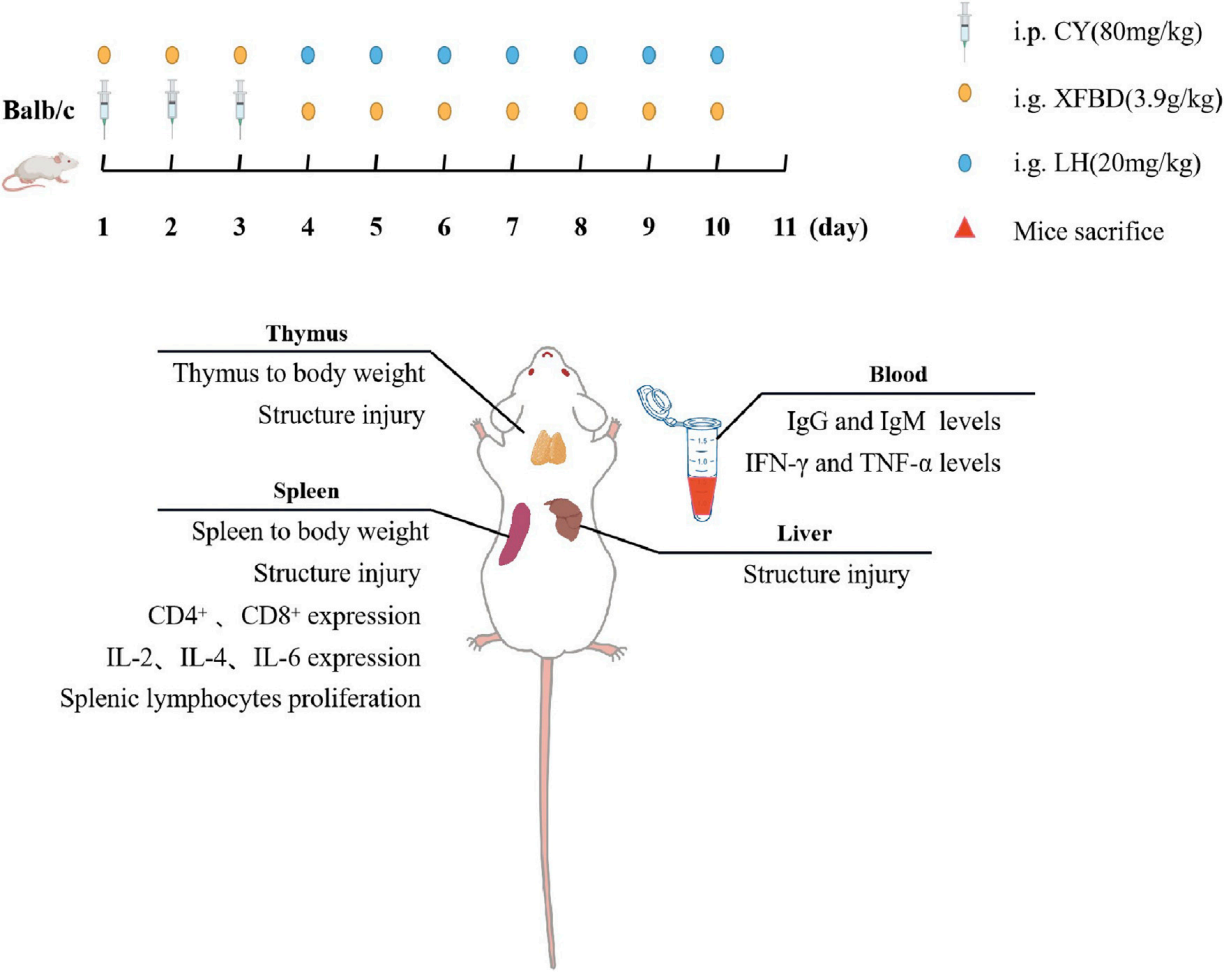


## Introduction

Cyclophosphamide (CY) is an alkylating agent that inhibits both the humoral and cellular immune responses (Emadi et al., 2009). It is widely used as a chemotherapeutic drug in the treatment of various types of diseases including cancer, autoimmune diseases, and organ transplants (Bensinger et al., 2001; Mamounas et al., 2005; Passweg and Tyndall, 2007). More importantly, the immunosuppressive state is one of the typical symptoms for a series of diseases such as coronavirus disease 2019 (COVID-19) caused by severe acute respiratory syndrome coronavirus 2 (SARS-CoV-2). It has been observed that the number of total T cells, CD4^+^, and CD8^+^ T cells was dramatically reduced in COVID-19 patients, especially in patients requiring intensive care (Diao et al., 2020). Therefore, many attempts are being made to obtain immune enhancement.

Xuanfei Baidu decoction (XFBD) was designed by Academician Boli Zhang and Professor Qingquan Liu for COVID-19 treatment as one of the clinical prescriptions, based on the recording of the famous traditional Chinese medicine book titled *Treatise on Febrile and Miscellaneous Diseases* (Pan et al., 2020). As the instruction described, XFBD prescripts 13 herbs, including *Ephedrae Herba*, *Armeniacae Semen*, *Gypsum Fibrosum*, *Coicis Semen*, *Atractylodis Rhizoma*, *Pogostemonis Herba*, *Artemisiae Annuae Herba*, *Polygoni cuspidati Rhizoma*, *Verbenae Herba*, *Phragmitis Rhizoma*, *Lepidii/Descurainiae Semen*, *Citri grandis Exocarpium Rubrum*, and *Glycyrrhizae Radix* (Wang et al., 2020). A previous study has analyzed the chemical composition of XFBD by HPLC-MS and HPLC-DAD-ESI-MS/MS (data unpublished). It has also been reported that XFBD in combination with conventional medicine significantly improved COVID-19 patient’s clinical symptoms, increased the number of white blood cells and lymphocytes, and reduced C-reactive protein and erythrocyte sedimentation rate, indicating a potential immune regulatory effect of XFBD in certain diseases (Xiong et al., 2020). Recently, our experimental data showed that XFBD ameliorated LPS-induced lung fibrosis *via* alleviating inflammatory reaction and attenuated staphylococcal protein A-induced inflammation in mice (data unpublished). However, the immunomodulatory effect of XFBD on immunosuppressed mice remains unknown. Here, we elucidated the protective effect of XFBD on immunosuppression in CY-treated mice, which may help provide evidence of XFBD for its clinical application.

## Materials and Methods

### Reagents

Lipopolysaccharide (LPS) was provided by Biological Industries (Shanghai, China). Injectable cyclophosphamide was obtained from Shanxi Pude Medicine Co., Ltd (Shanxi, China). Anti-CD4^+^ (FITC) and anti-CD8^+^ (APC) antibodies were provided by Bio Legend, Inc. (San Diego, CA, United States). The IgG and IgM kits were purchased from CLOUD-CLONE Co., Ltd (Wuhan, China). ELISA kits for cytokine measurement were purchased from Boaotuoda Biotechnology Co., Ltd (Beijing, China). The total RNA extraction kit was provided by YEASEN Co., Ltd (Shanghai, China). cDNA synthesis and qPCR kits were provided by Transgen (Beijing, China). Levamisole hydrochloride (LH) was produced from Renhe Church Pharmaceutical Co., Ltd (Shandong, China). The cell counting kit (CCK-8) was purchased from Invigentech, Inc. (Irvine, CA, United States).

### Extract of XFBD

XFBD was provided by the TianJin Modern TCM Innovation Center (TRT 200302) in the form of a freeze-dried powder. To prepare the XFBD extract, the freeze-dried powder of XFBD (0.4000 g) was extracted with ultrapure water (1:25, g/mL) in an ultrasonic water bath for 30 min. The solution was diluted with 50% methanol at the ratio of 1:1 and vortex-mixed for 5 min. Then the solution was centrifuged at 14,000 rpm for 10 min before filtered with a 0.22 μm filter membrane. An aliquot (2 μL) of the supernatant solution was injected into UHPLC-PDA for analysis. A Waters Acquity UHPLC System (Waters Co., Milford, MA) equipped with a photodiode array detector (PDA) was used to separate the multiple components in XFBD. All separations were performed using a ZORBAX RRHD Eclipse XDB-C18 column (2.1 × 100 mm, 1.8 μm, Agilent Technologies). The flow rate was 0.3 ml/min. The column temperature was 40°C. The mobile phase comprised (A) aqueous formic acid (0.1%, v/v) and (B) acetonitrile using a gradient elution of 5–10% B at 0–8 min, 10–15% B at 8–13 min, 15–17% B at 13–18 min, 17–45% B at 18–30 min, and 45–95% B at 30–35 min and the re-equilibration time of gradient elution was 5 min. The detection wavelength was set at 210 and 254 nm. The components in XFBD was investigated by UPLC and the contents including 1.19 mg/g for ephedrine, 4.97 mg/g for amygdalin, 3.63 mg/g for sinapine, 5.04 mg/g for hastatoside, 4.49 mg/g for verbenalin, 8.45 mg/g for polydatin, 3.40 mg/g for liquiritin, 3.46 mg/g for acteoside, 54.91 mg/g for naringin, and 4.80 mg/g for glycyrrhizic acid in the freeze-dried powder of XFBD (data not shown).

### Animals

Male BALB/c mice of 6–8 weeks old were purchased from the Charles River Laboratories (Beijing, China. License number: SCXK 2016-0006). The mice were housed under a 12 h light/dark cycle and were provided food and water. All experimental procedures were approved by the Animal Care and Use Committee of Tianjin University of Traditional Chinese Medicine (Animal Ethics Committee approval number: TCM-LAEC2020090).

### Cyclophosphamide-Induced Immunosuppression Model

Mice were randomly assigned to four groups (*n* = 6): the normal group (Normal), the immunosuppression model group (Model), the XFBD group (XFBD), and levamisole hydrochloride group (LH) as positive control. From day 1 to 3, mice in the normal control group were treated once daily with sterile saline, mice from the other three groups were intraperitoneally administered with cyclophosphamide at 80 mg/kg. From day 4 to 10, mice were given with the following treatment by gavage: the XFBD group with XFBD at 3.9 g/kg based on the conversion from an equivalent dose for patients in clinic (Xiong et al., 2020); the LH group with LH at 20 mg/kg; and the other two groups with water. Body weights were recorded every other day.

### Calculation of Immune Organ Index

Mice were sacrificed 24 h after last intragastric administration, and then the thymus and the spleen of each mouse were aseptically removed and weighed. The spleen and thymus indexes were calculated by the following formula: Spleen index or Thymus index (%) = (spleen or thymus weight/body weight) × 100%.

### Hematoxylin and Eosin Staining

After isolated from mice, the spleen, liver, and thymus tissues were fixed with 4% paraformaldehyde, embedded in paraffin and cut into 4 µm sections. Then, these sections were deparaffinized and stained with hematoxylin and eosin. Finally, the slices were then observed under a light microscope.

### Determination of Immunoglobulin and Cytokines in Serum by ELISA

The whole blood was obtained from mice killed by extracting eyeball under sterile conditions on the day of sacrifice. Blood samples were centrifuged at 2,500 rpm for 20 min at 4°C to obtain serum. The levels of immunoglobulin (IgG and IgM) and cytokine IFN-γ and TNF-α in the serum were measured according to the ELISA kit instructions.

### Lymphocyte Proliferation

The extirpated spleens obtained from the mice were washed with cold PBS and passed through a 200-mesh filter to obtain a homogeneous splenocyte suspension. The erythrocytes were lysed and the remaining cells were centrifuged at 1,000 rpm for 8 min. After centrifugation, cells were resuspended in RPMI-1640 medium with 20% FBS. Cells were placed into 96-well plates in triplicate at a density of 5×10^6^ cells/ml, and LPS (2 μg/ml) was added and followed by incubation for 48 h at 37°C in a humidified atmosphere containing 5% CO_2_. Then CCK-8 was added to each well, and after additional incubation for 2 h the absorbance at 450 nm was detected by a microplate reader.

### Determination of CD4^+^ and CD8^+^ T Lymphocytes in the Spleen

Spleen lymphocytes were first prepared as described above and were resuspended in PBS and adjusted to 1 × 10^7^/ml. Cell suspensions per 100 µL were incubated with anti-CD4^+^ (FITC) 0.5 µL and anti-CD8^+^ (APC) 1.25 µL for 30 min at 4°C under dark. The spleen lymphocytes were washed for two times with PBS. Then the ratio of CD4^+^ and CD8^+^ T lymphocyte subpopulations was analyzed by flow cytometry (BD FACS Calibur, United States).

### RNA Extraction and qRT-PCR Analysis

Total RNA of spleens was isolated by TRIzol (CWBIO, China). The RNA purity was detected by spectrophotometry. Then, RNA was reversely transcribed into cDNA according to a reverse transcription kit (Transgen Biotech, China). Then 20 µL PCR mixture containing 1 µL cDNA, 8 µL RNase-free water, 0.5 µL forward, 0.5 µL reverse primers, and 10 µL 2x qPCR Master Mix were prepared and a PCR reaction was then conducted on a real-time PCR system (BIO-RAD, California, United States). Each sample was tested three times. The relative gene expression profiles were determined by normalizing the expression to that of the reference gene (GAPDH) using the 2−ΔΔCt method. The primer sequences are shown in Table1.

**TABLE 1:** Primer sequences used for RT-PCR.

Gene	Forward primer (5′-3′)	Reverse primer (5′-3′)
*IL-2*	AGG​AAC​CTG​AAA​CTC​CCC​AG	AAA​TCC​AGA​ACA​TGC​CGC​AG
*IL-4*	TCT​CGA​ATG​TAC​CAG​GAG​CC	ACC​TTG​GAA​GCC​CTA​CAG​AC
*IL-6*	CTG​CAA​GAG​ACT​TCC​ATC​CAG	AGT​GGT​ATA​GAC​AGG​TCT​GTT​GG
GAPDH	AGG​TCG​GTG​TGA​ACG​GAT​TTG	TGT​AGA​CCA​TGT​AGT​TGA​GGT​CA

### Statistical Analysis

Data were shown as mean ± SD. The statistical significance of the differences between various groups was determined by an ANOVA analysis for multiple comparisons by Prism version 8.0. Differences between groups at *p* < 0.05 were considered statistically significant.

## Results

### XFBD Suppressed the Reduction of Body Weight and Immune Organ Index in CY-Induced Immunosuppression Mice

**FIGURE 1:**
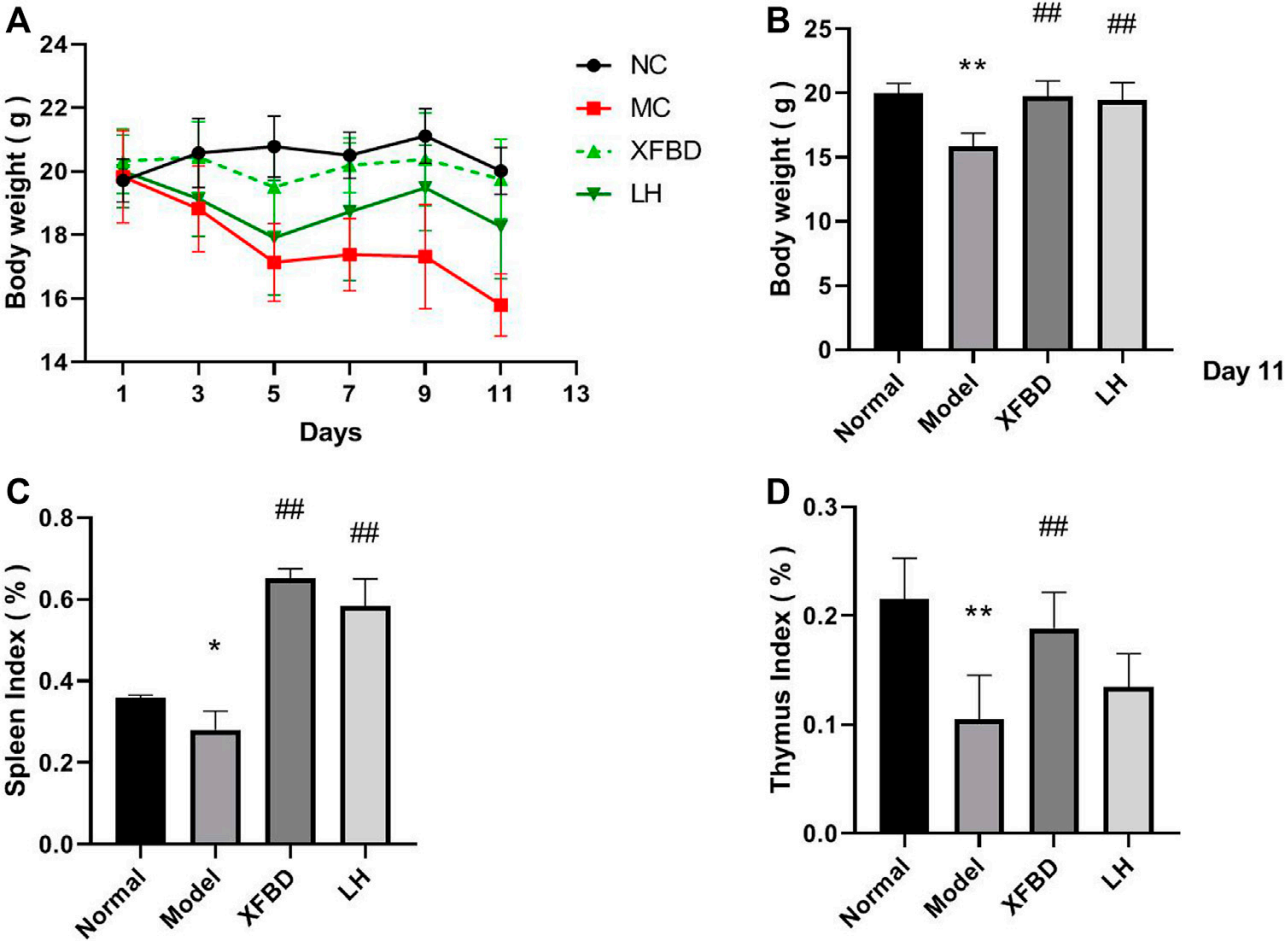
Effect of XFBD on body weights and immune organ indices. **(A)** Body weights during treatment; **(B)** body weights after treatment; **(C)** spleen index, and **(D)** thymus index. Normal, administered with saline; Model: intraperitoneally administered with cyclophosphamide; XFBD: intraperitoneally administered with cyclophosphamide first and followed by XFBD at 3.9 g/kg/day; and LH: intraperitoneally administered with cyclophosphamide first and followed by LH at 20 mg/kg. Data are expressed as mean ± SD (*n* = 6).*p < 0.05 and **p < 0.01 vs. Normal group, ^#^ *p* < 0.05 and ^##^ *p* < 0.01 vs. Model group.

To investigate the immune modulation of XFBD, CY-induced immunosuppression model was established in mice and there was a remarkable decrease of body weight in the Model group after injection of CY during the therapeutic 11 days. Administration with XFBD significantly rescued the weight lost (Figure 1A), which was most obviously observed on day 11 (Figure 1B). In addition, treatment with LH resulted in a similar effect as that of XFBD in body weight. Furthermore, both of the decreased spleen index and the thymus index were observed in the Model group when compared with those in the Normal group (Figures 1C,D) as we expected. However, after administration with XFBD, the spleen and the thymus indices were highly enhanced when compared with those in the Model group and both of which were even better than those in the LH group.

### XFBD Improved the Pathological Alterations of the Liver, Spleen, and Thymus in CY-Induced Immunosuppression Mice

**FIGURE 2:**
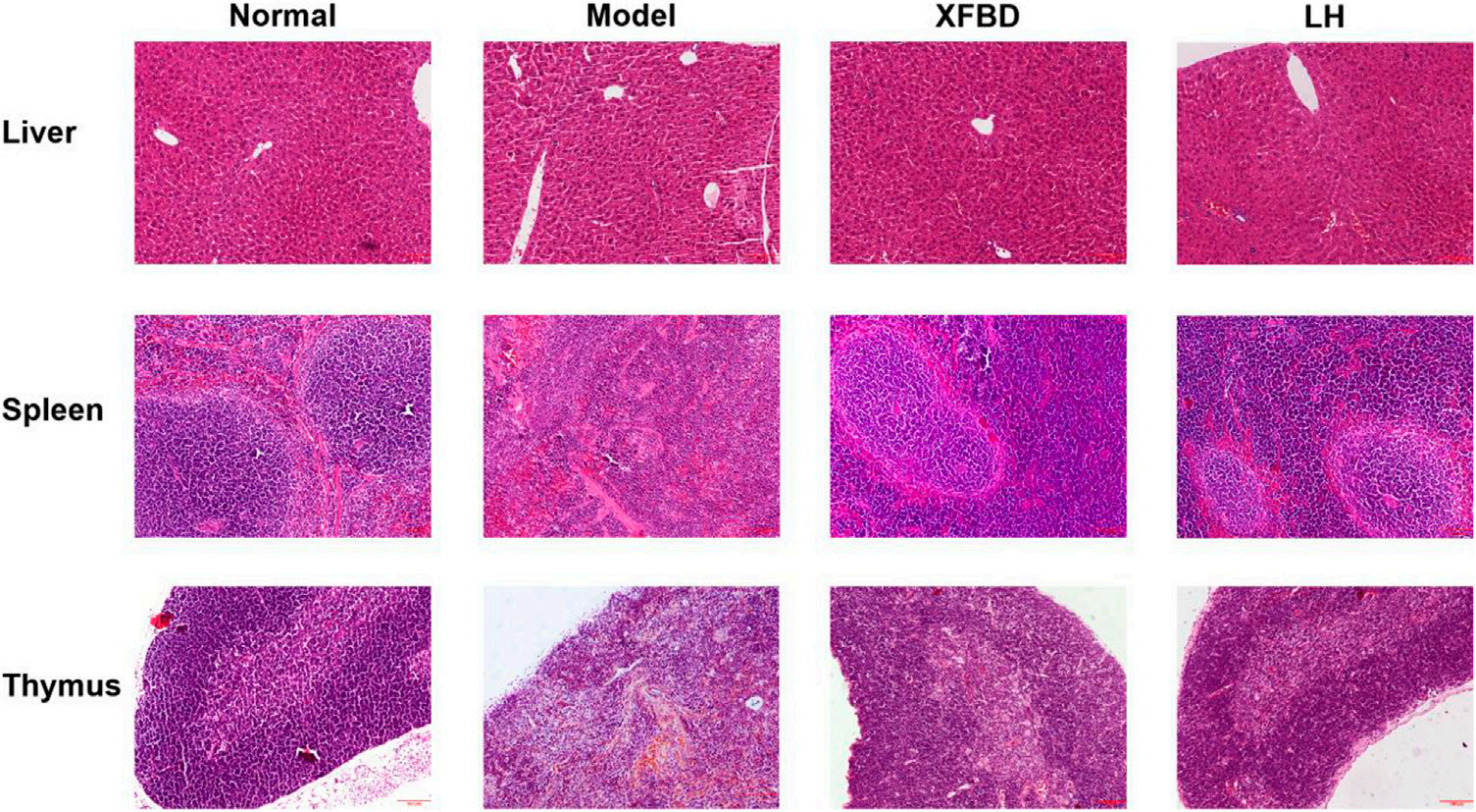
Effects of XFBD on the morphological changes in the liver, the spleen, and the thymus in CY-treated mice. stained by H&E, 10× Normal, administered with saline; Model: intraperitoneally administered with cyclophosphamide; XFBD: intraperitoneally administered with cyclophosphamide first and followed by XFBD at 3.9 g/kg/day; and LH: intraperitoneally administered with cyclophosphamide first and followed by LH at 20 mg/kg.

A pathological analysis by HE staining showed that the arrangement of liver sinusoid and hepatocyte cords in the Normal group were destroyed and massive inflammatory cells were infiltrated in CY-induced immunosuppression mice (Figure 2, liver). In the spleen, white pulp atrophy, hemorrhage, and necrosis were easily observed in the Model group, and red pulp and white red pulp were intermixed as well when compared with those in the Normal group (Figure 2, spleen). In the thymus, the clear tissue structures including cortex, medulla, and thymic corpuscle were observed in the Normal group, which were much destroyed in the Model group (Figure 2, thymus). However, XFBD administration significantly inhibited the pathological alterations in the mouse liver, spleen, and thymus induced by CY. Besides, as the positive control, the pathological alteration of the LH group was also much rescued, similar to those in the XFBD group (Figure 2).

### XFBD Enhanced the Serum Levels of Cytokines and Immunoglobulin in CY-Induced Immunosuppression Mice

**FIGURE 3:**
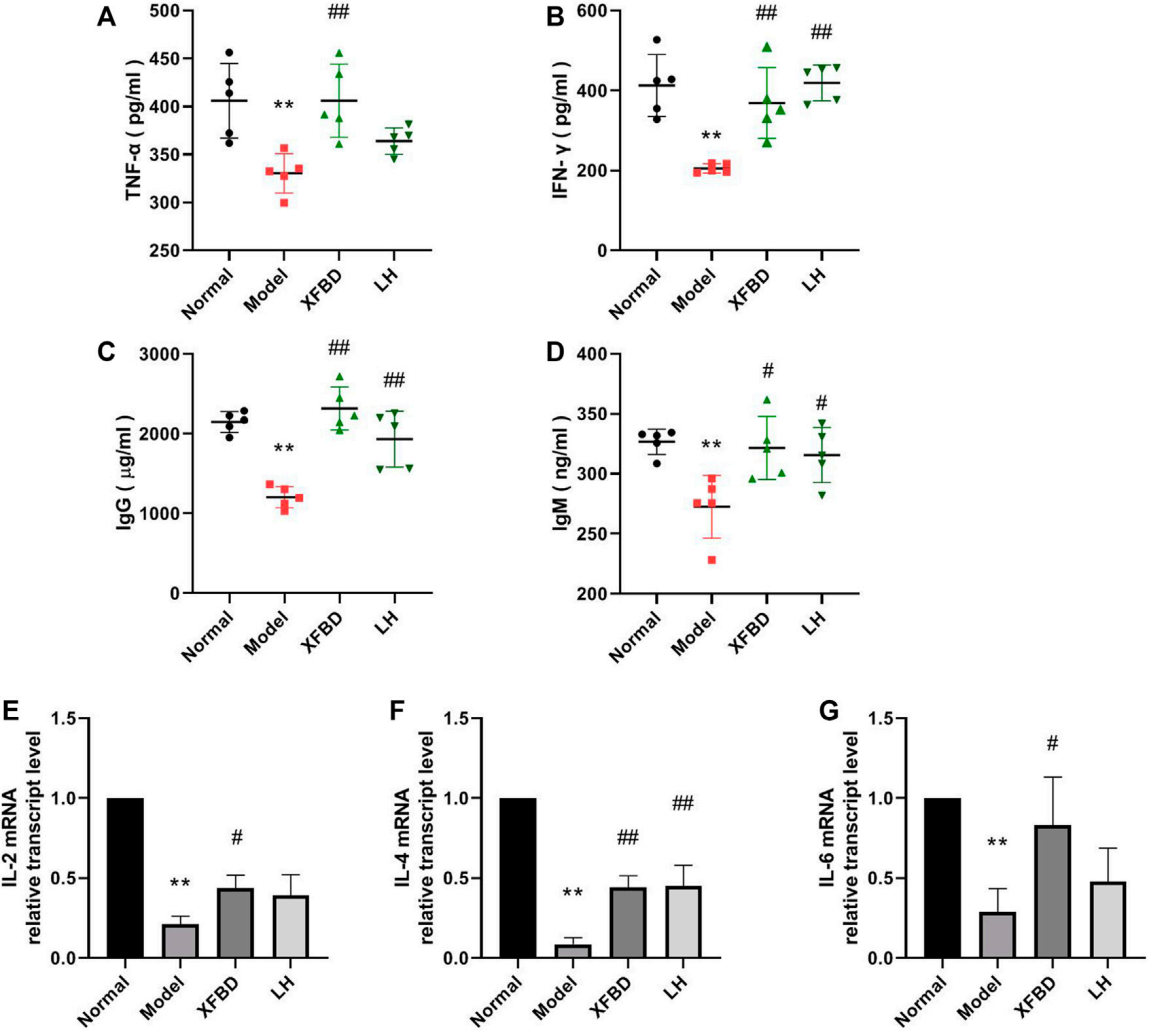
Effect of XFBD on immunoglobulin levels and cytokines in the CY-treated mice. TNF-α **(A)**, IFN-γ **(B)** IgG **(C)**, and IgM **(D)** levels in serum were determined by ELISA and IL-2 **(E)**, IL-4 **(F)**, and IL-6 **(G)** expressions in the spleen were determined by RT-PCR. Normal, administered with saline; Model: intraperitoneally administered with cyclophosphamide; XFBD: intraperitoneally administered with cyclophosphamide first and followed by XFBD at 3.9 g/kg/day; and LH: intraperitoneally administered with cyclophosphamide first and followed by LH at 20 mg/kg. Data are expressed as mean ± SD [**(A–D)**, *n* = 5] [**(E–F)**, *n* = 3]. *p < 0.05 and **p < 0.01 vs. Normal group, #*p* < 0.05 and ##*p* < 0.01 vs. Model group.

CY injection caused significant reduction of cytokine TNF-α and IFN-γ levels in serum, which were further rescued by XFBD (Figures 3A,B). Similarly, we found that the concentrations of IgG and IgM in serum were noticeably decreased in the Model group when compared with that in the Normal control group but highly increased in the XFBD group (Figures 3C,D). The effects of XFBD on promotion of cytokines and immunoglobulin were even better than those of LH.

### XFBD Alleviated the Reduction of Cytokine Expression in Spleen Isolated From CY-Induced Immunosuppression Mice

The relative gene expressions of IL-2, IL-4, and IL-6 in the mouse spleen were determined by RT-PCR. Data showed that CY significantly decreased the expressions of IL-2, IL-4, and IL-6, which were rescued after XFBD or LH treatment (Figures 3E–G).

### XFBD Promoted Proliferation of Splenic Lymphocytes Isolated From CY-Induced Immunosuppression Mice in Response to LPS

**FIGURE 4:**
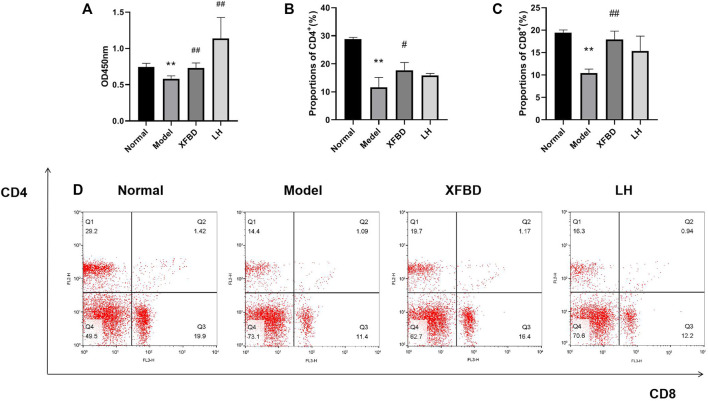
Effects of XFBD on LPS-induced splenic lymphocyte proliferation and T lymphocyte subsets in CY-treated mice. **(A)** LPS-induced splenic lymphocyte proliferation; **(B)** representative flow cytometry analysis result of CD4^+^ subset; **(C)** representative flow cytometry analysis result of CD8^+^ subset; and **(D)** representative images of Splenic T lymphocyte subsets detected by flow cytometry. Normal, administered with saline; Model: intraperitoneally administered with cyclophosphamide; XFBD: intraperitoneally administered with cyclophosphamide first and followed by XFBD at 3.9 g/kg/day; and LH: intraperitoneally administered with cyclophosphamide first and followed by LH at 20 mg/kg. Data are expressed as mean ± SD (*n* = 3).*p < 0.05 and **p < 0.01 vs. Normal group, #*p* < 0.05 and ##*p* < 0.01 vs. Model group.

Splenic lymphocytes were isolated from different groups of mice and LPS-stimulated cell proliferation was measured. As shown in Figure 4A, LPS-induced splenic lymphocytes proliferation was significantly reduced in the Model group, when compared with that in the Normal group; however, it was further enhanced after treatment with XFBD.

### XFBD Induced Splenic Lymphocytes of CD4^+^ and CD8^+^ T Cells in CY-Induced Immunosuppression Mice

The number of CD4^+^ and CD8^+^ T lymphocytes in the spleen was evaluated by flow cytometry. The percentages of CD4^+^ and CD8^+^ T lymphocytes in the Model group were significantly lower than those in the Normal group. However, the reduction of CD4^+^ and CD8^+^ T lymphocytes were then increased in XFBD groups, indicating an effect of XFBD on immune promotion, which was consistently observed in the LH group (Figures 4B–D).

## Discussion

Coronavirus disease 2019 (COVID-19) broke out in 2019 and spread rapidly around the world, causing a global pandemic (Li et al., 2020). Up to now, the National Health Commission of China has published eight versions of diagnosis and treatment guidelines (National Health Commission National Administration of Traditional Chinese Medicine, 2020) for COVID-19 therapy. Traditional Chinese medicine (TCM) has a history of more than 2,000 years in prevention and treatment of epidemics and plagues (Ren et al., 2020). By the systemic diagnosis from TCM experts, it has been generally believed that COVID-19 is a pestilence in view of its epidemic and infectious nature, characterized with the external evils such as toxin evil, wet evil, and cold evil (Zheng et al., 2021), and therefore, COVID-19 is classified as “wet toxin pestilence.”

According to the previous reports, lymphopenia is a key feature of patients with COVID-19, especially in severe cases. Patients with severe COVID-19 are more likely to exhibit lymphopenia on admission, resulted in a broad immune cell reduction including CD4^+^ and CD8^+^ T cells, NK cells, and monocytes and dendritic cells. (Yang et al., 2020; Xu et al., 2020; Zhou et al., 2020). As far as we known, during SARS-CoV-2 infections, the virus appears to first immunosuppress the host and then taking advantage of the body’s natural system (Jacques and Apedaile, 2020). It is well known that CY is a common chemotherapeutic drug and administration of CY leads to immunosuppression (Ahlmann and Hempel, 2016; Wang et al., 2011) such as killing immune cells, interfering with the proliferation and differentiation of B and T cells, and restraining the humoral and cellular immune response (Sistigu et al., 2011; Zhu et al., 1987; Shirani et al., 2015). Consistently, in our study, we found that CY caused weight loss of the immune organs (Figure 1), the reduction levels of cytokines in serum and the spleen (Figures 3A,B,E–G), and the imbalance of IgG and IgM production in the peripheral blood of mice (Figures 3C,D), which eventually inhibited the immune function (Noh et al., 2019). It is worthy to notice that COVID-19 links between immunosuppression and hyperinflammation (Jacques and Apedaile, 2020). The first stage is immunosuppression characterized by lymphopenia combined with T-cell exhaustion and an inadequate adaptive immune response, aimed to evade the immune system allowing for unchecked viremia. It has been found that the presence of SARS-CoV-2 virus induced T-cell lymphopenia and T-cell exhaustion (Diao et al., 2020). The second stage in severe SARS-CoV-2 infection is hyperinflammation characterized by a cytokine storm with neutrophil, monocyte/macrophage infiltration, and activation. The infected patients show increased level of cytokines, chemokines, increased numbers of neutrophils, and monocytes. In our study, we used CY-induced immunosuppressive mice as an animal model mimic the immunosuppressive state observed in COVID-19 patients. However, we did not evaluate the effect of XFBD in the second hyperinflammatory stage. Other available models such as acute lung injury and the pulmonary fibrosis model, or SARS-CoV-2–infected hACE2 mice need to be included to investigate the anti–COVID-19 effects in pneumonia, viral replication, multiple organ failure, and even death in future research.

XFBD was first created by Academician Boli Zhang and Professor Qingquan Liu, and it is highly recommended by the National Administration of Traditional Chinses Medicine concerning its definite clinical effect for COVID-19 patients as an immune modulator drug. In March 2021, XFBD got the drug approvement by China Drug Administration (Zhang et al., 2020). In particular, the toxicity of XFBD has been evaluated at the dose of 142.8 g/kg. The only appearance of prone and decreased movement was observed 1–4 h after administration of XFBD, while female mice showed soft or loose stool for 1–3 days after administration of XFBD (142.8 g/kg). No other abnormality such as body weight and anatomic characteristics was found in groups. Therefore, we thought that the maximum tolerated dose of XFBD given to mice shall not be less than 142.8 g/kg.

In our study, we first found that XFBD has an immunoregulatory role in CY-induced immunosuppression mice. Data showed that compared to the Model group, the thymus and the spleen indices of XFBD-treated groups were significantly increased (Figure 1), the pathological alteration in the liver, the spleen, and the thymus were much improved (Figure 2), and splenic CD4^+^ and CD8^+^ T cells and LPS-induced lymphocyte proliferation were also highly increased (Figure 4). In cytokine expression, XFBD was able to significantly reverse the decline of serum TNF-α and IFN-γ and mRNA of spleen IL-2, IL-4, and IL-6 in CY-treated mice (Figure 3). Taken together, our results gave evidence that XFBD has multiple positive effects to enhance cellular immunity by regulating component of immune systems in CY-treated mice. For cellular assay, the anti-inflammatory effect of XFBD has also been investigated in LPS-induced THP-1 monocytes and RAW 264.7 macrophages. As we expected, XFBD significantly inhibited LPS-induced IL-6, IP-10, and TNF-α expressions in THP-1 cells (data not shown) and TNF-α, IL-6, and IL-1β secretions in RAW 264.7 cells (data not shown), indicating an immune-modulatory effect of XFBD in a bidirectional manner.

Previously, our group employed the network strategy to predict the potential signaling pathways of XFBD which may be involved in the anti–COVID-19 effect (Wang et al., 2011). Data showed that the main biological pathways regulated by XFBD included viral infection, energy metabolism, immunity and inflammation, and parasites and bacterial infections, which indicated a multi-herb, multi-constituent, and multi-target pattern, with lung as the chief targeted organ for therapy of XFBD in COVID-19. More efforts needed to make to further uncover the mechanism of XFBD in immunomodulation.

Taken together, our data first showed that XFBD might be involved in immunomodulatory effects *in vivo*, providing an evidence of basis use of XFBD for the prevention and treatment of immunosuppressive diseases, and also indicating the potential role of XFBD for COVID-19 treatment.

## Data Availability

The original contributions presented in the study are included in the article/Supplementary Material; further inquiries can be directed to the corresponding authors.

## Abbreviations

XFBD, Xuanfei Baidu decoction; CY, cyclophosphamide; TCM, traditional Chinese medicine; COVID-19, coronavirus disease 2019; SARS-CoV-2, severe acute respiratory syndrome coronavirus 2; LPS, lipopolysaccharide; LH, levamisole hydrochloride; CCK-8, cell counting kit; PDA, photodiode array detector; HE, hematoxylin and eosin; GAPDH, glyceraldehyde-3-phosphate dehydrogenase; TNF-α, tumor necrosis factor-α; IFN-γ, interferon-γ; IgG, immunoglobulin G; IgM, immunoglobulin M; IL-2, interleukin-2; IL-4, interleukin-4; IL-6, interleukin-6.
