# Corrigendum: Prevention of Cyclophosphamide-Induced Immunosuppression in Mice With Traditional Chinese Medicine Xuanfei Baidu Decoction

**DOI:** 10.3389/fphar.2021.808424

**Published:** 2022-01-20

**Authors:** Huimin Yan, Jia Lu, Jiabao Wang, Lu Chen, Yu Wang, Lin Li, Lin Miao, Han Zhang

**Affiliations:** ^1^ State Key Laboratory of Component-based Chinese Medicine, Tianjin University of Traditional Chinese Medicine, Tianjin, China; ^2^ Institute of Traditional Chinese Medicine, Tianjin University of Traditional Chinese Medicine, Tianjin, China; ^3^ Key Laboratory of Pharmacology of Traditional Chinese Medical Formulae, Ministry of Education, Tianjin University of Traditional Chinese Medicine, Tianjin, China

**Keywords:** immunosuppression, immune modification, cyclophosphamide, traditional Chinese medicine (TCM), Xuanfei Baidu decoction (XFBD)

In the original article, there was a mistake in Table 1 as published. We would like to correct “Primer sequences used for RT-PCR Table, as errors were introduced in the preparation of this table for publication.” The corrected Table 1 appears below.

**TABLE 1:** Primer sequences used for RT-PCR.

Gene	Forward primer (5′-3′)	Reverse primer (5′-3′)
*IL-2*	AGG​AAC​CTG​AAA​CTC​CCC​AG	AAA​TCC​AGA​ACA​TGC​CGC​AG
*IL-4*	TCT​CGA​ATG​TAC​CAG​GAG​CC	ACC​TTG​GAA​GCC​CTA​CAG​AC
*IL-6*	CTG​CAA​GAG​ACT​TCC​ATC​CAG	AGT​GGT​ATA​GAC​AGG​TCT​GTT​GG
GAPDH	AGG​TCG​GTG​TGA​ACG​GAT​TTG	TGT​AGA​CCA​TGT​AGT​TGA​GGT​CA

The authors apologize for this error and state that this does not change the scientific conclusions of the article in any way. The original article has been updated.

